# Aerobic and Anaerobic Speed Predicts 800-m Running Performance in Young Recreational Runners

**DOI:** 10.3389/fphys.2021.672141

**Published:** 2021-05-21

**Authors:** Øyvind Støren, Jan Helgerud, Jan-Michael Johansen, Lars-Erik Gjerløw, Aanund Aamlid, Eva Maria Støa

**Affiliations:** ^1^Department of Natural Sciences and Environmental Health, University of South-Eastern Norway, Bø, Norway; ^2^Department of Sports, Physical Education and Outdoor Studies, University of South-Eastern Norway, Bø, Norway; ^3^Department of Circulation and Medical Imaging, Norwegian University of Science and Technology, Trondheim, Norway; ^4^Myworkout, Medical Rehabilitation Centre, Trondheim, Norway

**Keywords:** middle-distance running, sprint performance, time to exhaustion, aerobic power, anaerobic speed reserve

## Abstract

The main aim was to investigate the impact of maximal aerobic speed (MAS), maximal anaerobic speed (MANS), and time to exhaustion (TTE) at 130% MAS, on 800-m running time performance (800TT). A second aim was to investigate the impact of anaerobic speed reserve (ASR), i.e., the relative difference between MAS and MANS, on TTE. A total of 22 healthy students classified as recreational runners participated in a cross-sectional study. They were tested for maximal oxygen consumption (VO_2max_), oxygen cost of running (C_R_), time performance at 100 m (100TT), time performance at 800 m (800TT), and TTE. MAS was calculated as VO_2max_ × C_R_^–1^, and MANS was calculated as 100TT velocity. Both MAS and MANS correlated individually with 800TT (*r* = –0.74 and –0.67, respectively, *p* < 0.01), and the product of MAS and MANS correlated strongly (*r* = –0.82, *p* < 0.01) with 800TT. TTE did not correlate with 800TT. Both ASR and % MANS correlated strongly with TTE (*r* = 0.90 and –0.90, respectively, *p* < 0.01). These results showed that 800TT was first and foremost dependent on MAS and MANS, and with no impact from TTE. It seemed that TTE was merely a product of each runner’s individual ASR. We suggest a simplified model of testing and training for 800TT, namely, by focusing on VO_2max_, C_R_, and short sprint velocity, i.e., MAS and MANS.

## Introduction

Middle-distance running such as the 800 m puts great demands on both aerobic and anaerobic ATP production (Spencer and Gastin, 2001; Duffield et al., 2005). Ingham et al. (2008) using allometric models have found maximal aerobic speed (MAS), calculated as maximal oxygen uptake (VO_2max_) divided by the oxygen cost of running (C_R_), to predict a large proportion of 800-m time performance (800TT). As maximal sprint velocity is the highest running velocity obtainable with maximal anaerobic energy release, it may be termed maximal anaerobic speed (MANS), being the anaerobic equivalent to MAS. Several previous studies have established a relationship between 800TT and MAS or VO_2max_ (Boileau et al., 1982; Brandon and Boileau, 1992; Camus, 1992; Nevill et al., 2008), but few have established the relationship between 800TT and MANS. However, Bachero-Mena et al. (2017) found correlations between 20-m sprint and 800TT, and between 200 m and 800TT. In addition, Sandford et al. (2019a) have put focus on the role of anaerobic speed reserve (ASR), i.e., the difference between MAS and MANS, as an important performance determining factor in the 800 m. However, from investigating the role of ASR in 1500-m runners, Sandford et al. (2019b) highlights an important principle, that ASR in isolation is not indicative of an athlete’s caliber if not put in context with the actual level of MAS and MANS. Ortiz et al. (2018) have demonstrated a positive correlation between MANS and ASR, but a negative correlation between MAS and ASR in soccer players.

The ratio of aerobic/anaerobic energy reliance during the 800 m naturally increases with increasing race time, from approximately a 60/40% distribution in runners using less than 120 s to approximately a 70/30% distribution for runners using more than 150 s (Medbø and Tabata, 1989; Duffield et al., 2005; Nevill et al., 2008). Several studies have therefore focused on the role of anaerobic capacity for 800 m, measured as mean accumulated oxygen deficit (MAOD). The term anaerobic capacity in this sense is, however, somewhat misleading, as it does not differ between maximal anaerobic capacity measured in, e.g., a single jump or power lift, and the ability to sustain a certain amount of anaerobic energy release over time. We propose to use anaerobic endurance capacity as a better measure for this purpose. MAOD is often measured over 2 min to exhaustion, following the protocol by Medbø et al. (1988). The results from previous MAOD studies are equivocal regarding the impact on 800TT. Craig and Morgan (1998) found no significant relationship between MAOD and 800TT. Ramsbottom et al. (1994) and Billat et al. (2009) found that higher MAOD correlated with better 800TT, while Tanji et al. (2018) found that lower MAOD correlated with better 800TT. Nevill et al. (2008) combined aerobic and anaerobic capacity in the equation 0.223MAOD.0.818VO_2m__*ax*_ and found this product to correlate strongly with 800TT.

The use of MAOD for evaluating anaerobic endurance has the advantage of indirectly quantifying the amount of anaerobic energy release. However, this method has several disadvantages. Firstly, if MAOD is calculated within a set time to exhaustion (TTE) (e.g., 2 min), different athletes may work at different relative intensities (e.g., 120–140% MAS, 65–90% MANS, or 20–60% ASR). Secondly, if MAOD is calculated within a set intensity (e.g., 120% MAS), the TTE may differ (e.g., 60–180 s). Thirdly, MAOD is a complex test to accomplish, and with some discomfort for the participants as it would imply running to exhaustion on the treadmill. According to Hill and Vingren (2011), the same MAOD applied whether the runners were running to exhaustion in either 3, 5, or 7 min. This may indicate an individual set maximal amount of anaerobic energy release for each runner in a continuous middle distance event. A consequence of this may be that what could be termed anaerobic endurance is just a matter of portioning out an anaerobic capacity. If so, at a given supramaximal intensity in relation to MAS, the runner working at the lowest percentage of his or her MANS would use the least of the anaerobic capacity per unit of time—and endure the longest. In relation to this assumption, Billat et al. (2009) proposed a model saying that any instant, running speed should be controlled by the prevailing anaerobic store remaining. Blondel et al. (2001) reported that TTE at 120 and 140% of MAS correlated strongly with ASR. Based on these results, they concluded that the same intensity relative to MAS did not represent the same absolute intensity for all and proposed to express intensity as a percentage of ASR for supra-maximal velocities. On the other hand, if TTE was to be measured at a given percentage of ASR, a logical proposition is that the runner with the lowest percentage of MANS still would endure the longest. In addition, if a runner has a narrow gap between MAS and MANS, a given percentage of ASR would imply a higher relative usage of MAS compared with a runner with a broader gap between MAS and MANS. It could thus be argued against using both MAOD, TTE at a percentage of MANS, or TTE a percentage of ASR as valid measures of anaerobic endurance. It could of course also be argued against using TTE at a given percentage of MAS as a valid measure of anaerobic endurance, given the arguments raised in Blondel et al. (2001). However, the same relative supramaximal intensity relative to MAS at least ensures the same relative VO_2_ demand. We therefore propose to simply measure TTE at a set supramaximal intensity, e.g., 130% MAS. The 130% MAS is chosen based on the previous work by Blondel et al. (2001), measuring TTE at 120 and 140% of MAS. The downside of such a test is that it does not quantify the amount of anaerobic energy release. The pros of such a test is that it may be performed on a track and that it ensures the same relative intensity for all runners given the knowledge of each runner’s MAS. If two runners perform to exhaustion at the same relative supramaximal intensity, the runner performing the longest time should logically display the highest relative VO_2_ deficiency, although it might still be debatable if this would be a valid test for measuring anaerobic endurance.

Predicting 800-m performance based on physiological measures is therefore a complex task. There seems to be a scientific gap related to how much of 800TT that may be explained by MAS, MANS, ASR, and anaerobic endurance capacity. Gaining more knowledge on this could be important for choices on how to train. The aim of the present study was thus to investigate the relative impact of MAS, MANS, and TTE at 130% MAS on 800-m time performance, and further to investigate the impact of ASR on TTE. The hypothesis was that 800-m time performance would be determined by MAS and MANS, but not TTE, and that TTE would be determined by ASR.

## Materials and Methods

### Experimental Approach

In order to investigate the impact of MAS, MANS, and TTE on 800-m time performance (800TT), and to evaluate the impact of ASR on TTE, a cross-sectional study was performed. This study included testing of VO_2max_, C_R_, 100-m time performance (100TT), 800TT, and TTE at 130% MAS. Comparisons regarding all selected variables were then made between men and women, between the fastest and slowest in 800TT, and between those with the highest and lowest ASR. In addition, correlation analyses were performed between 800TT and the other variables, and between TTE and the other variables.

### Subjects

A total of 22 healthy students (14 males and 8 females) participated in the present study. The participants’ characteristics are presented in Table 1. All participants engaged regularly in sports like recreational running competitions (e.g., 3k and 10k), soccer, or volleyball. In addition, all were running regularly as a training modality. However, none of them was competing in middle-distance running. Although with longer performance times in the 800TT than 800-m specialists, the performance times are still representative for an all-out supramaximal exercise relative to VO_2max_ in middle distances, i.e., 2–4 min maximal running.

**TABLE 1:** General characteristics of participants.

	**All (*N* = 22)**	**Males (*N* = 14)**	**Females (*N* = 8)**	**TT < 160 s (*N* = 9)**	**TT > 160 s (*N* = 13)**	**ASR > 175% (*N* = 11)**	**ASR < 175% (*N* = 11)**
Age (yrs)	21.8 ± 3.9 (17.9)	21.8 ± 2.9 (13.3)	21.8 ± 5.5 (25.1)	22.1 ± 2.8 (12.7)	21.5 ± 4.6 (21.4)	20.5 ± 2.4 (11.7)	23.1 ± 4.7 (20.3)
Height (cm)	176.6 ± 8.3 (4.7)	182.2 ± 5.7 (3.1)	168.6 ± 5.4 (3.2)**	181.8 ± 6.8 (3.7)	173.0 ± 7.5 (4.3)^##^	178.0 ± 8.3 (4.7)	175.2 ± 8.5 (4.9)
BW (kg)	77.3 ± 14.3 (18.5)	83.3 ± 13.7 (16.4)	66.8 ± 8.4 (12.6)**	78.5 ± 8.3 (10.6)	76.5 ± 17.7 (23.1)	79.7 ± 18.7 (22.8)	74.9 ± 8.4 (11.2)
800mTT (s)	167.1 ± 18.2 (10.9)	158.3 ± 13.9 (8.8)	182.4 ± 14.4 (7.9)**	149.8 ± 5.1 (3.4)	179.0 ± 13.5 (7.5)^##^	173.5 ± 19.5 (11.2)	160.6 ± 14.9 (9.3)

Values are mean ± standard deviation, with coefficient of variance in percent in parenthesis. yrs, years. cm, centimeters. kg, kilograms. s, seconds. m, meters. TT, 800-m time trial. **p < 0.01 significantly different from males. ^##^p < 0.01 significantly different from 800 TT < 160 s.

The study was approved by the Institutional Research Board at the University of South-Eastern Norway and the Norwegian Centre for Research Data (NSD, reg. 183455) and conducted in accordance with the Helsinki declaration. All participants gave their written consent to participate, after having received information about the study.

### Testing

The participants were tested on four different days. The first 2 days were performed on two consecutive days, while days 3–4 came within 2 weeks from the first test day. Day 1 consisted of C_R_ and VO_2max_ measurements in the laboratory. From these results, MAS was calculated as VO_2max_ × C_R_^–1^. Day 2 consisted of 100TT and 800TT at the University’s athletic outdoor track. Since most of the participants were unacquainted with competing at 800 m, and thus might misjudge the pacing according to their own capacity, a second 800TT was performed at day 4. Day 3 consisted of TTE at 130% MAS on the outdoor athletic track.

C_R_ and VO_2max_ were tested by use of the metabolic test system, MetaLyzer II Cortex (Biophysic GmbH, Leipzig, Germany). The O_2_ analyzer was calibrated with ambient air and certified calibration gases (16% O_2_/4% CO_2_), while the flow sensors were calibrated with a 3-L calibration syringe (Biophysic GmbH, Leipzig, Germany) before each test. Both tests were performed on a Woodway PPS 55 sport (Waukesha, WI, United States), calibrated for speed and incline. HR was registered with Polar s610 HR monitors (Kempele, Finland).

The C_R_ measurements were performed as an integrated part of the warm-up before the VO_2max_-test and performed as flat treadmill running. Since C_R_ was primarily used to calculate MAS, C_R_ had to be measured at an aerobic intensity with minimal anaerobic contribution. After 5 min of easy jogging at 60–70% of HR_max_, the participants ran 5 min with at least the three last minutes at steady state at 80% of HR_max_. Steady state was defined as maximum 1 ml increase or decrease in VO_2_ from the median of three subsequent registrations and, accordingly, maximum 1 beat per minute regarding HR. With a submaximal steady state, these measurements should not be biased by individual VO_2_ slow components. This intensity corresponds to a VO_2_ inside 70–90% VO_2max_. Based on Helgerud et al. (2010), this intensity range will give the same C_R_, which was calculated as VO_2_ × *v*^–1^, and expressed as ml × kg*^–^*^1^×m*^–^*^1^.

The VO_2max_ test was an incremental test to voluntary exhaustion performed at 5% inclination, as previously used in Støren et al. (2008) and Helgerud et al. (2010). The participants started at an intensity predicted to be approximately 80% of HR_max_. Speed was increased by 0.5 km × h^–1^ every 30 s. All participants were instructed to run to voluntary fatigue, and the three highest successive VO_2_ measurements were used to calculate VO_2max_. As previously used in Helgerud et al. (2010) and Støa et al. (2020), HR > 95% of HR_max_, respiratory exchange ratio (RER) > 1.05, and flattening of the VO_2_ curve (corresponding to not more than 1-ml increase in VO_2_ in three subsequent registrations at the end of the test) were used as criteria to evaluate if VO_2max_ was obtained.

On the second test day, 100TT was tested after a standard warm up of 10–15 min easy run and three to five short runs with gradually increasing speed up to approximately maximal sprint velocity. All 100TTs were performed on the straight of the running track with the wind behind. A maximum wind speed of 2.0 m × s^–1^ was permitted. A 10–15-min active pause with easy jogging followed the 100TT test and preceded the 800TT test. The participants were tested in heats of two persons in 100TT and three to six persons in 800TT to simulate a competition setting. For both the 100TT and the 800TT, all tests were performed in stay weather, with temperatures between 12 and 17°C.

Previous studies have assessed MANS by flying short sprints in order to measure the actual top speed (Blondel et al., 2001; Buchheit et al., 2012; Ortiz et al., 2018; Julio et al., 2020; Selmi et al., 2020). In the present study, a standard 100-m sprint was used to assess this variable. The rationales for this choice were that a standard 100-m sprint would be more easy to access by the general coach or athlete and that the results can be compared to race results as was done with the results from the open-access Norwegian database in the present study.

On the third test day, the TTE at 130% MAS was performed on the same outdoor track, paced by a cyclist. The 130% MAS was chosen based on the previous work by Blondel et al. (2001), measuring TTE at 120 and 140% of MAS. The intensity had to be supramaximal relative to VO_2max_, but low enough to ensure that it would represent anaerobic endurance and not MANS. A cycle with a cycle computer was calibrated against the laboratory treadmill to ensure the correct velocity being displayed. Both the runner and the cyclist accelerated for approximately 30 m, and passed the starting point at the right velocity. The runner was to keep 1–2 m distance from the cycle—and the test terminated when the distance exceeded 2 m. Timers were spread around the track, to get the best possible view for when the participants would fall too far behind the cycle. The average velocity was checked by dividing distance covered by time taken.

ASR was calculated as the difference between MANS and MAS and presented as both in relative values, as a percentage of MAS, and in absolute values in km × h^–1^. ASR relative to MAS was chosen since absolute values can be misleading when comparing fast and slow runners and thus men and women.

The 800TT is highly dependent of pacing (Thiel et al., 2012). Since the subjects were unacquainted with 800-m races and could have misjudged the pacing, a second 800TT was performed 1–2 weeks after the first one. By adding a second test, the participants were able to adjust the pace if they had started the first race too hard or too carefully. Prior to this second attempt, the participants received advice to either increase or decrease their starting velocity, dependent on whether or not they had a too hard or too slow start in the first race. In addition, this second attempt was without a preceding 100TT, leaving out a possible deterioration of the 800TT because of this. Of the 22 participants, 15 improved their 800TT in their second attempt. The best 800TT result for each runner was used for the analyses regardless of being the first or second attempt.

In order to compare MANS and the impact of MANS on 800TT with a larger Norwegian data material, the open-access Norwegian athletics results database (friidrett.no) was searched. All athletes from 16 years and older and with registered outdoor results for both 100 and 800 m the same season, i.e., 2019, were used. The 69 athletes’ results were then analyzed for correlations.

### Statistics

Sample size was calculated by expected differences in VO_2max_ between the fastest and slowest runners. With a mean of 50 ml × kg^–1^ × min^–1^, a difference of 10 ml⋅kg^–1^⋅min^–1^, a standard deviation of the same size as the difference, a power of 80%, and a significance level of 0.05, we would need 16 participants. Given a possible dropout of approximately 20% and a level of uncertainty, we recruited 23 participants, of whom one withdrew from the study. For the 22 participants, data were tested by use of the Shapiro-Wilk, and by QQ plots for 800TT and for TTE at 130% MAS, and found to represent normal distributions. Descriptive data are therefore presented as mean ± standard deviation (SD). To further present variability, the coefficients of variance (CV) are presented as percent in order to show a common denomination for all variables. Independent sample *t* tests were used to compare men and women, to compare the fastest and slowest runners, and to compare those with the highest and lowest ASR. The fastest and slowest runners and the runners with the highest and lowest relative ASR were divided into groups over or under mean result, and not by median. By dividing the participants into these groups, it was possible to display what characterizes the fastest runners and the runners with the highest ASR. In order to evaluate possible relationships between 800TT or TTE at 130% MAS and the other variables, Pearson’s bivariate correlation test was used. Standard error of the estimate (SEE) was applied in all correlations from linear regression analyzes. The SEE values were then converted into percent values by dividing them by the variable means and then multiplied by 100. Correlations were performed including all participants, but to check for the possible impact of gender, all correlations were repeated corrected for gender in partial correlation analyses. As gender showed little impact on the correlations with one exception addressed in the *Discussion*, only the uncorrected correlations are presented in the *Results* section. In order to address the possible overlap, i.e., co-linearity regarding MAS and MANS relations to 800TT, variation inflation factor (VIF) and tolerance tests were performed. To further address predictability of 800TT results based on MAS, MANS, and TTE, multiple regressions were performed as MAS × MANS vs. 800TT and as MAS × MANS × TTE vs. 800TT. For the 69 athletes’ results obtained from the Norwegian open-access database, normal distribution was found by use of the same method as for the 22 main participants in the present study. Pearson’s bivariate correlation test was then performed in order to check the possible correlation between 100 and 800TT in this group. The Statistical Package for Social Science version 26 (SPSS, IBM, Chicago, IL, United States) was used for all statistical analyses performed. A *p* < 0.05 was taken as the level of significance in all tests (two-tailed).

## Results

Characteristics of participants and comparisons between male and females, the fastest and slowest runners, and the runners with the highest and the lowest ASR are presented in Tables 1, 2.

**TABLE 2:** Performance and physiological results.

	**All (*N* = 22)**	**Males (*N* = 14)**	**Females (*N* = 8)**	**TT < 160 s (*N* = 9)**	**TT > 160 s (*N* = 13)**	**ASR > 175% (*N* = 11)**	**ASR < 175% (*N* = 11)**
800mTT (s)	167.1 ± 18.2 (10.9)	158.3 ± 13.9 (8.8)	182.4 ± 14.4 (7.9)**	149.8 ± 5.1 (3.4)	179.0 ± 13.5 (7.5)^##^	173.5 ± 19.5 (11.2)	160.6 ± 14.9 (9.3)
TTE (s)	88.6 ± 39.4 (44.5)	93.9 ± 40.8 (43.5)	79.4 ± 37.7 (47.5)	84.0 ± 35.1 (41.8)	91.8 ± 43.3 (47.2)	116.7 ± 35.8 (30.7)	60.6 ± 15.9 (26.2)^§§^
100mTT (s)	13.6 ± 1.2 (8.8)	13.0 ± 1.0 (7.7)	14.8 ± 0.8 (5.4)**	12.6 ± 0.6 (4.8)	14.4 ± 1.0 (6.9)^##^	13.8 ± 1.0 (7.2)	13.5 ± 1.5 (3.7)
MANS (km × h^–1^)	26.6 ± 2.4 (9.0)	27.8 ± 1.9 (6.8)	24.5 ± 1.3 (5.3)**	28.7 ± 1.3 (4.5)	25.2 ± 1.8 (7.1)^##^	26.2 ± 1.8 (6.9)	27.0 ± 2.9 (10.7)
MAS (km × h^–1^)	15.2 ± 1.9 (12.5)	15.8 ± 2.0 (12.7)	14.3 ± 1.5 (10.5)	16.6 ± 1.6 (9.6)	14.3 ± 1.6 (11.2)^##^	13.8 ± 1.2 (8.7)	16.7 ± 1.8 (10.8)^§§^
Pred800TT (s)	162.5 ± 20.3 (12.5)	154.1 ± 19.7 (12.8)	177.1 ± 11.6 (6.5)**	142.1 ± 10.6 (7.5)	175.9 ± 13.1 (7.5)^##^	175.0 ± 16.6 (9.5)	150.0 ± 15.7 (10.5)^§§^
ASR (% MAS)	176.4 ± 19.9 (11.3)	178.0 ± 16.4 (9.2)	173.5 ± 25.9 (14.9)	174.0 ± 30.9 (17.7)	178.0 ± 54.9 (30.8)	191.4 ± 14.7 (7.7)	161.3 ± 10.8 (6.7)^§§^
ASR (km × h^–1^)	11.4 ± 2.1 (18.4)	12.0 ± 1.4 (11.7)	10.1 ± 2.6 (25.7)	12.1 ± 1.1 (9.0)	10.9 ± 2.4 (22.0)	12.5 ± 1.4 (11.2)	10.3 ± 2.1 (20.4)
VO_2m__*ax*_ (ml × kg^–1^ × min^–1^)	54.9 ± 6.1 (11.1)	56.8 ± 6.3 (11.1)	51.6 ± 4.3 (8.3)*	60.8 ± 2.4 (3.9)	50.8 ± 4.2 (8.3)^##^	53.2 ± 6.0 (11.3)	56.6 ± 6.0 (10.6)
C_*R*_ (ml × kg^–1^ × m^–1^)	0.218 ± 0.024 (11.0)	0.217 ± 0.022 (10.1)	0.219 ± 0.028 (12.8)	0.222 ± 0.024 (10.8)	0.215 ± 0.024 (11.2)	0.232 ± 0.023 (10.0)	0.203 ± 0.013 (6.4)^§§^

Values are mean ± standard deviation, with coefficient of variance in percent in parenthesis. s, seconds. m, meters. TT, 800-m time trial. TTE, time to exhaustion at 130% MAS. MAS, maximal aerobic speed (VO_2max_/C_*R*_). MANS, maximal anaerobic speed (from 100 m TT). km × h^–1^, kilometers per hour. VO_2max_ (ml × kg^–1^ × min^–1^), maximal oxygen uptake in milliliters per kilogram body mass per minute. C_*R*_ (ml × kg^–1^ × m^–1^), oxygen cost of running in milliliters per kilogram body mass per meter. Pred800TT, predicted velocity at 800 m and is calculated from 0.2MANS + 0.8MAS for the runner using more than 160 s, and 0.3MANS + 0.7MAS for the runners using less than 160 s. ASR, anaerobic speed reserve in percent above MAS or in km × h^–1^. *p < 0.05 significantly different from males. **p < 0.01 significantly different from males. ^##^p < 0.01 significantly different from 800 TT < 160 s. ^§§^p < 0.01 significantly different from ASR > 75%.

The participants ran the 800 m in 167.1 ± 18.2 s, i.e., a mean race time of 2 min 47 s. The mean velocity was thus 17.2 km × h^–1^. With a mean 100-m velocity of 26.6 km × h^–1^, they ran the 800 m at 64.7% of MANS.

Mean VO_2_ demand for the 800TT was 63.2 ± 9.4 ml × kg^−1^ × min^–1^, which was 115% of VO_2m__*ax*_. The 800TT velocity of 17.2 km.h^–1^ was accordingly 113% of the MAS velocity of 15.2 km × h^–1^.

The predicted 800TT results based on 0.2MANS + 0.8 MAS for the slowest runners (>160 s) and 0.3MANS + 0.7MAS for the fastest runners (<160 s) and the measured 800TT results correlated strongly (*r* = 0.87, *p* < 0.001). The VIF for possible overlap between MAS and MANS was 1.4, with a collinearity tolerance of 0.7.

The males were 8% taller and 25% heavier than the females. They had 13% faster 800TT and 12% faster 100TT. Both VO_2max_ and MAS were 10% higher in the males than in the females. There were no significant differences between males and females in age, TTE, ASR, or C_R_.

The fastest 800-m runners were 16% faster than the slowest runners were. They were 5% taller, 12% faster in the 100TT, had 16% higher MAS, and 20% higher VO_2m__*ax*_ than the slowest runners. There were no significant differences between the fastest and slowest runners in age, body weight (BW), TTE, ASR, or C_R_.

The runners with the highest ASR had 30% points higher ASR than those with the lowest ASR, representing a 2.2 km.h^–1^ difference in absolute terms. They had 93% longer TTE, 17% lower MAS, and 14% higher C_R_ than the runners with the lowest ASR. There were no significant differences between those with the highest and those with the lowest ASR in age, height, BW, 800TT, 100TT, or VO_2max_.

**FIGURE 1:**
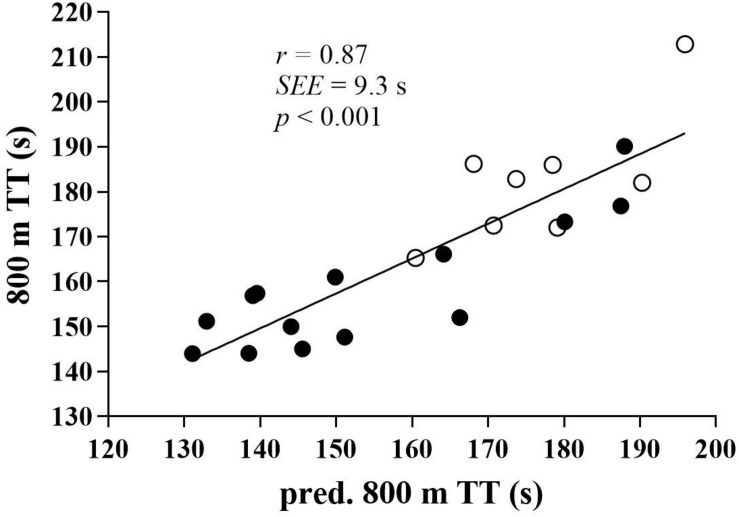
The relationship between predicted and measured 800TT. Values are predicted 800TT (time trial) in seconds (s) on the *x*-axis and measured 800TT (s) on the *y*-axis. Males are denoted in black circles, and females are indicated in white circles. The correlation is statistically significant (*p* < 0.001). *r*, correlation coefficient. SEE, standard error of the estimate.

**FIGURE 2:**
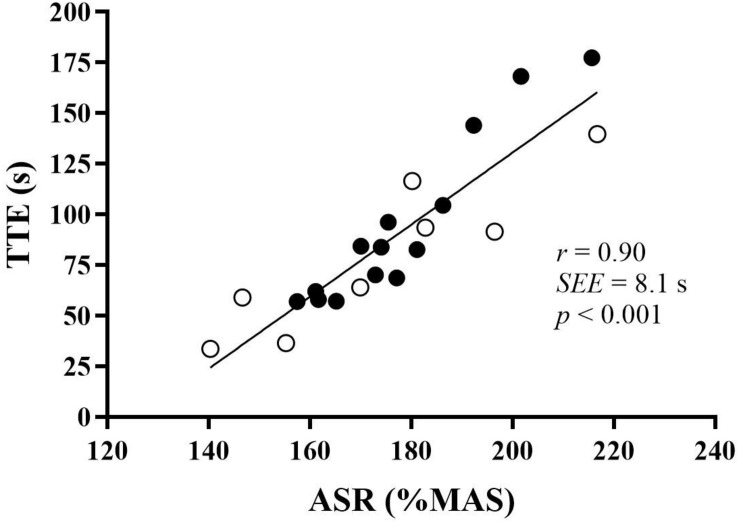
The relationship between ASR and TTE at 130% MAS. Values are ASR (anaerobic speed reserve) in percent (%) on the *x*-axis and TTE (time to exhaustion) in seconds (s) on the *y*-axis. Males are denoted in black circles, and females are indicated in white circles. The correlation is statistically significant (*p* < 0.001). *r*, correlation coefficient. SEE, standard error of the estimate. MAS, maximal aerobic speed.

Correlations with 800TT and TTE at 130% MAS are presented in Tables 3, 4 and in Figures 1, 2.

**TABLE 3:** Correlations with 800TT and TTE, independent of gender.

	**Correlations with 800-m TT (*N* = 22)**			**Correlations with TTE (*N* = 22)**		
	***r***	***p***	**SEE (%)**	***r***	***p***	**SEE (%)**
800-m TT (s)	−	−	−	0.19	0.389	44.8
TTE (s)	0.19	0.389	11.3	−	−	−
100-m TT (s)	0.65	0.001**	8.5	−0.14	0.549	44.9
MANS (km × h^–1^)	−0.67	0.001**	8.3	0.10	0.687	45.4
MAS (km × h^–1^)	−0.74	0.000**	7.5	−0.69	0.000*	32.7
Pred800TT (s)	0.87	0.000**	5.5	0.46	0.030*	40.3
ASR (%)	−0.35	0.116	10.5	0.90	0.000**	20.1
ASR (km × h^–1^)	−0.08	0.720	11.2	0.76	0.000**	30.3
VO_2m__*ax*_ (ml × kg^–1^ × min^–1^)	−0.74	0.000**	7.5	−0.13	0.653	44.8
C_*R*_ (ml × kg^–1^ × m^–1^)	0.11	0.639	11,1	0.69	0.000**	32.7

Values are the correlation coefficient r, the significance level p, and the standard error of the estimate (SEE) in percent (%). s, seconds. m, meters. TT, time trial. TTU, time to exhaustion at 130% MAS. MAS, maximal aerobic speed (VO_2max_/C_*R*_). MANS, maximal anaerobic speed (from 100 m TT). km × h^–1^, kilometers per hour. VO_2max_ (ml × kg^–1^ × min^–1^), maximal oxygen uptake in milliliters per kilogram body mass per minute. C_*R*_ (ml × kg^–1^ × m^–1^), oxygen cost of running in milliliters per kilogram body mass per meter. Pred800TT, predicted velocity at 800 m and is calculated from 0.2MANS + 0.8MAS for the runner using more than 160 s, and 0.3MANS + 0.7MAS for the runners using less than 160 s. ASR, anaerobic speed reserve in percent above MAS or in km × h^–1^. *p < 0.05 significant correlation. **p < 0.01 significant correlation.>

**TABLE 4:** Partial correlations with 800TT and TTE, corrected for gender.

	**With 800-m TT (*N* = 22)**		**With TTE (*N* = 22)**	
	***r***	***p***	***r***	***p***
800-m TT (s)	–	–	0.41	0.066
Pred800TT (s)	0.80	0.000*	−0.65	0.001*
TTE (s)	0.41	0.066	–	–
MANS (km × h^–1^)	−0.39	0.082	0.05	0.842
MAS (km × h^–1^)	−0.70	0.000*	−0.81	0.000*
ASR (%)	0.45	0.060	0.92	0.000*
ASR (km × h^–1^)	0.31	0.171	0.82	0.000*
VO_2m__*ax*_ (ml × kg^–1^ × min^–1^)	−0.68	0.001	−0.15	0.66
C_*R*_ (ml × kg^–1^ × m^–1^)	−0.15	0.520	0.76	0.000*

Values are the correlation coefficient r and the significance level p. s, seconds. m, meters. TT, time trial. TTE, time to exhaustion at 130% MAS. MAS, maximal aerobic speed (VO_2max_/C_*R*_). MANS, maximal anaerobic speed (from 100 m TT). km × h^–1^, kilometers per hour. Pred800TT, predicted velocity at 800 and is calculated from 0.2MANS + 0.8MAS for the runner using more than 160 s, and 0.3MANS + 0.7MAS for the runners using less than 160 s. ASR, anaerobic speed reserve in percent above MAS or in km × h^–1^. *p < 0.01 significant correlation.

The 800TT correlated negatively with MAS and MANS (*r* = –0.74 and –0.67, respectively, *p* < 0.01), meaning that the higher MAS or MANS, the faster 800TT. The multiple regression MAS × MANS vs. 800TT gave an *r* value of –0.81 (*p* < 0.01). Neither TTE nor ASR correlated significantly with 800TT. TTE correlated positively with ASR, meaning that the higher ASR, the longer TTE. TTE correlated negatively with MAS, meaning that the higher MAS, the shorter TTE. C_R_ correlated positively with TTE, meaning that the higher C_R_, the longer TTE (Tables 2, 3). The multiple regression MAS × MANS × TTE vs. 800TT gave an *r* value of –0.82 (*p* < 0.01). A multiple regression for MAS × MANS × ASR vs. 800TT gave an *r* value of –0.83 (*p* < 0.01), when ASR was expressed in relative values and –0.80 when ASR was expressed in absolute values.

The 800TT was run at 61.2 ± 14.9% of ASR, and the TTE at 130% MAS was run at 74.6 ± 14.9% of MANS. The latter percentage also correlated strongly with TTE (*r* = 0.90, *p* < 0.001).

The interpretation of open-access results obtained from the Norwegian database (friidrett.no) on 69 runners with both 100-m and 800-m results in 2019 revealed a moderate correlation (*r* = 0.69, *p* < 0.01) between 100TT and 800TT. Of the 69 runners, there were 37 males and 32 females, with a mean age of 20.3 ± 4.0 years. Mean 100TT was 12.6 ± 1.0 s, and mean 800TT was 131.9 ± 1.0 s.

## Discussion

The main finding of the present study was that performance in 800TT was determined by MAS and MANS, but not by TTE at 130% MAS. This means that primarily MAS and sprint ability determined 800-m performance. TTE at 130% MAS was determined by ASR and by the percentage of MANS, both showing that those running at lowest percentage of their sprinting capacity could endure the longest. These patterns were the same in the male and the female runners. The present results thus confirmed our initial hypothesis.

VO_2max_ revealed a strong correlation with 800TT in the present study (*r* = –0.74, *p* < 0.01). This is in accordance with Boileau et al. (1982); Brandon and Boileau (1992), Camus (1992), and Nevill et al. (2008). However, and in contrast to the present study, neither Craig and Morgan (1998) or Tanji et al. (2018) found VO_2max_ to be predicative for 800TT. A possible reason for the latter may be that the participants in the present study were more heterogeneous regarding VO_2max_ than those in Craig and Morgan (1998) or Tanji et al. (2018). The 100TT correlated moderately with 800TT in the present study (*r* = –0.65, *p* < 0.01). This is in accordance with the interpretation of open-access results from Norwegian Athletics Association, 2019. (friidrett.no), revealing a very similar relationship (*r* = 0.69, *p* < 0.01) when all athletes above 16 years of age that competed in both 100 m and 800 m in the same season are taken together. The present results are also in accordance with Bachero-Mena et al. (2017) who found correlations between 20-m sprint and 800-m time performance (*r* = 0.72, *p* < 0.01) and between 200 and 800-m time performance (*r* = 0.84, *p* < 0.01).

The predicted 800TT results were based on 0.2MANS + 0.8 MAS for the slowest runners (>160 s) and 0.3MANS + 0.7MAS for the fastest runners (<160 s). Although we were underestimating real values by approximately 5 s, the predicted and the measured 800TT results correlated strongly (*r* = 0.87, *p* < 0.001). Indirectly, these results are in agreement with the 0.223MAOD × 0.818VO_2max_ equation proposed by Nevill et al. (2008) in runners with approximately 30 s better performance times in 800TT. The sum of prediction *r-*values for MANS and MAS, the *r*^2^-values, equaled 1.0 in the present study. Given no overlap between MAS and MANS, these two variables should statistically explain the whole time performance in the 800TT. A VIF of 1.4 and a collinearity tolerance of 0.7 indicate very little overlap between MAS and MANS. A multiple regression analysis of MAS × MANS vs. 800TT revealed an *r* value of 0.81, whereas MAS × MANS × TTE vs. 800TT only increased *r* to 0.82. When comparing the fastest with the slowest runners (Table 1), the fastest runners had higher MAS and MANS, but approximately similar TTE. As also C_R_ was similar between the fastest and the slowest runners (Table 1), in short, the runners with the highest VO_2max_ and the fastest 100-m times performed best at 800 m. These results thus support our initial hypothesis that 800-m time performance would be determined by MAS and MANS, but not TTE. TTE at 130% MAS did not correlate with 800TT. Actually, when looking behind the significance level, it was the slowest runners at 800 m who exhibited the longest TTE. Whether or not TTE at 130% MAS is a valid measure of anaerobic endurance is debatable. TTE seemed to be determined largely by ASR. The correlation between these two variables was very strong in both relative terms (*r* = 0.90, *p* < 0.001) and in absolute terms (*r* = 0.76, *p* < 0.001). This makes sense as it was the slowest runners at 800 m who had the lowest MAS, and those with the lowest MAS had the largest ASR (Table 1). The correlation is in accordance with results from Blondel et al. (2001), finding strong correlations between ASR and TTE at 120 and 140% of MAS (*r* = –0.83, *p* < 0.01 and *r* = –0.94, *p* < 0.001, respectively). These results indicate that anaerobic endurance could be primarily determined by the relative ASR. This assumption was strengthened by calculating 130% MAS as a percentage of MANS. The present results exhibited the same as Blondel et al. (2001), that the same intensities relative to MAS was not the same relative to MANS. A strong correlation in the present study then revealed that those running at the lowest percentage of MANS at 130% MAS showed the longest time results at TTE (*r* = 0.90, *p* < 0.01). The present results thus further support an assumption that given a set individual amount of MAOD, those who can portion this out the best can endure the longest. The results further support the model proposed by Billat et al. (2009), that any instant running speed should be controlled by the prevailing anaerobic store remaining. A natural consequence of this is that maximal sprint velocity sets the potential for anaerobic endurance. The finding in the present study that TTE at 130% MAS did not correlate with 800TT performance, is thus indirectly in accordance with results from Craig and Morgan (1998), finding no significant relationship between MAOD and 800-m race time. The present results are in contrast to the significant correlations shown in Ramsbottom et al. (1994), *r* = –0.61 (*p* < 0.05) between MAOD measured at 120% MAS and 800-m race time. On the other hand, Tanji et al. (2018) actually found the opposite of Ramsbottom et al. (1994), namely, that the slowest 800-m runners had the highest MAOD, *r* = 0.70 (*p* < 0.01).

The finding in the present study that ASR was approximately similar between the fastest and the slowest runners was not surprising in light of the results from Sandford et al. (2019a,b). When finding that ASR was positively related to 800-m performance and negatively related to 1,500-m performance, Sandford et al. (2019b) stressed that ASR in isolation is not indicative of an athlete’s caliber. As in the present study, Sandford et al. (2019b) showed that with a higher MAS, but not a higher MANS, ASR becomes lower although the higher MAS in itself would contribute to a better performance. The present results that the runners with the highest ASR had the lowest MAS support the findings by Ortiz et al. (2018) who demonstrated a positive correlation between MANS and ASR, but a negative correlation between MAS and ASR in soccer players.

The patterns regarding determinants for the 800TT and the TTE were the same in the male and the female runners. The males had better 800TT, and higher MANS, only a tendency (*p* = 0.06) to better MAS, but similar TTE and ASR to the females (Table 1). When performing partial correlations correcting for gender, all significant correlations remained significant with the exception of MANS vs. 800TT, while all non-significant correlations remained non-significant. All *r*-values were approximately the same as for the uncorrected correlations.

### Practical Implications

From the present results, a natural suggestion for improving 800TT results is to focus on improving MAS and MANS. There could be a potential for simplifying the coaches approach to performance determining variables in 800 m running, by focusing solely on velocity. Any focus on improving anaerobic endurance may be excessive, if taking the assumption that it is merely a product of ASR into consideration. Maximal strength training has in previous studies been shown to both improve C_*R*_ (Støren et al., 2008) and sprint performance (Blagrove et al., 2018). Maximal strength training thus has the potential of improving both MAS and MANS. In addition, sprint training in distances up to 100 m has shown good effect on sprint running performance (Rumpf et al., 2016) and high-intensity aerobic interval training has shown very good effect on VO_2max_ (Helgerud et al., 2007). Based on this, we propose a combination of high-intensity aerobic interval training, short sprint training, and maximal strength training in order to improve 800TT.

### Limitations and Future Perspectives

We cannot completely rule out that the results regarding the relationships between TTE at 130% MAS and 800TT or ASR could have been different if the TTE test had been performed at 120 or 140% MAS. However, we have found no reasons as to why these relationships should be substantially different. With a TTE test at a lower intensity than 120% MAS, or a higher intensity than 140% MAS, the intensity would have been too close to either MAS or MANS, respectively, to be a good measure of anaerobic endurance.

In the 800TT tests, we simulated a competitive setting by using heats 3–6 participants simultaneously. While this may have inspired to maximal effort among the runners, it could also have had an impact on the runners’ pacing strategies.

The choice of using a standard 100TT to assess MAS and not flying short sprints has both pros and cons. A standard 100TT is more easy to access by the general coach or athlete, and the results can be compared to race results like the results from the open-access Norwegian database in the present study. However, although 100TT does represent MANS, it is not the exact top speed. Therefore, we cannot completely rule out that the inter-individual differences in 100TT could have been slightly different as the top speed is measured at the middle of the 100TT.

The present study was conducted with healthy students that may be characterized as recreational runners. In order to apply also for competitive or elite middle-distance runners, the study should be replicated with participants at the highest performance level possible. In addition, the correlations presented show relationships between variables, but do not necessarily represent causality. In order to investigate the cause–response relationships, intervention studies should be performed. Theoretically, if the present results do represent causality, an intervention improving MANS while maintaining MAS should then result in improvements in both 800TT and TTE at 130% MAS. An intervention improving MAS while maintaining MANS should then improve 800TT, but deteriorate TTE at 130% MAS.

## Conclusion

Performance in 800TT was determined by MAS and MANS, but not by TTE. TTE at 130% MAS was determined by ASR and by the percentage of MANS. We suggest training to improve VO_2max_, C_R_, and maximal sprint velocity as the most effective means to improve 800TT.

## Data Availability Statement

The raw data supporting the conclusions of this article will be made available by the authors, without undue reservation.

## Ethics Statement

The study was approved by the Institutional Research Board at the University of South-Eastern Norway and the Norwegian Centre for Research Data (NSD, reg. 183455). The patients/participants provided their written informed consent to participate in this study.

## Author Contributions

ØS, ES, and JH participated significantly in the planning and design of the study, as well as in data analysis and the writing of the manuscript. ØS, ES, JH, J-MJ, L-EG, and AA participated in the data collection, data analysis, and writing of the article. All authors read and approved the manuscript.

## Conflict of Interest

The authors declare that the research was conducted in the absence of any commercial or financial relationships that could be construed as a potential conflict of interest.

## Abbreviations

ASR: anaerobic speed reserve.
BW: body weight.
C_R_: oxygen cost of running.
MAOD: mean accumulated oxygen deficiency.
MAS: maximal aerobic speed.
MANS: maximal anaerobic speed.
TT: time trial.
TTE: time to exhaustion.
VO_2max_: maximal oxygen consumption.
